# Application of strain tomography and contour method to residual stress analysis in additively manufactured CM247LC superalloy parts

**DOI:** 10.1007/s40964-025-01116-2

**Published:** 2025-04-23

**Authors:** Fatih Uzun, Mohamed Fares Slim, Hector Basoalto, Konstantinos Liogas, Zifan Ivan Wang, Alexander M. Korsunsky

**Affiliations:** 1 University of Oxford, Oxford, UK; 2 Arts et Metiers Institute of Technology, Paris, France; 3 University of Sheffield, Sheffield, UK; 4 Diamond Light Source, Didcot, UK

**Keywords:** Residual stress reconstruction, Synchrotron X-ray diffraction, Elastic strain mapping, Finite element analysis, Laser powder bed fusion, High-temperature superalloys

## Abstract

Residual stresses are recognized as a critical factor influencing the mechanical performance and structural integrity of additively manufactured parts, particularly in nickel-based superalloys. Although the contour method and strain tomography have been applied independently for residual stress evaluation of such materials, a direct comparison of their reconstructions in laser powder bed fusion fabricated specimens has not been reported. In this study, both techniques were employed on identically produced specimens of CM247LC superalloy, and a strong qualitative agreement in residual elastic strain distributions was observed. Using the contour method, tensile residual stresses up to +1300 MPa were identified near the specimen edges, while compressive stresses approaching − 600 MPa were found in the central regions. Strain tomography, based on synchrotron X-ray diffraction, was used to non-destructively reconstruct internal residual elastic strain fields, revealing consistent trends and capturing localized variations aligned with the contour method. Through this integrated approach, a complete validation of stress reconstruction was achieved, and new insights into the stress evolution of laser powder bed fusion manufactured CM247LC were provided. The findings demonstrate how the complementary strengths of these techniques can be leveraged for improved residual stress characterization in high-performance superalloy parts.

## Introduction

Additive manufacturing [1] has transformed the production of parts that require high performance, particularly for applications requiring exceptional mechanical and thermal properties [2–4], such as those in the aerospace [5–10] and energy [11–15] industries. Among the various additive manufacturing techniques, laser powder bed fusion (LPBF) is distinguished by its ability to produce intricate geometries with high precision [16–19]. This process involves the selective melting of metal powders using a laser, layer by layer, enabling the fabrication of complex parts with tailored microstructures [20, 21]. The CM247LC nickel-based superalloy, renowned for its outstanding creep strength and oxidation resistance, has gained significant attention for LPBF applications due to its ability to meet the stringent requirements of turbine parts and other high-temperature environments [22, 23]. However, the inherent thermal gradients and rapid solidification associated with LPBF frequently lead to non-equilibrium microstructures and residual stresses, which can compromise the mechanical integrity and service performance of fabricated parts [24–28]. Addressing these challenges is essential for fully exploiting the benefits of LPBF for CM247LC superalloys.

Residual stresses are a critical concern in LPBF-fabricated parts, particularly for high-performance materials like the CM247LC superalloy [29–31]. These stresses influence mechanical properties such as fatigue resistance [32–35], dimensional stability [36], and fracture toughness, often limiting the applicability of LPBF-fabricated parts in safety-critical industries like aerospace [23, 37–39] and power generation [40, 41]. Accurate quantification of these residual stresses is essential for understanding their effects on part performance and optimizing process parameters to mitigate stress concentrations [42, 43]. However, the complexity of LPBF-produced parts, including their anisotropic microstructures [44, 45], high surface roughness [46], and intricate geometries [47–49], poses significant challenges for residual stress measurement. Conventional techniques, such as sin^2^psi X-ray diffraction [50–53] and hole-drilling [54–57], often struggle to capture the multiscale, two- or three-dimensional stress states present in these parts. Additionally, the hardness [58–60] and thermal stability [61, 62] of CM247LC and other superalloys complicate sample preparation and measurement using traditional methods, necessitating the development and application of advanced experimental techniques.

Residual stress reconstruction [63–72] is pivotal for understanding the mechanical performance and integrity of advanced materials like additively manufactured CM247LC superalloy [73]. Among the various methods available for residual stress analysis, the contour method [74–86] and strain tomography [87] are particularly notable for their complementary capabilities. The contour method is a destructive technique [88] that involves sectioning the material along a plane of interest, measuring the resultant surface deformation using high precision profilometry, and numerically modelling the stress field responsible for the observed deformation. This approach is highly effective for obtaining two-dimensional residual stress maps with high spatial resolution and accuracy [85, 89–91]. However, its destructive nature limits applicability to unique specimens and precludes further use of the sample for additional analyses and the method's success depends on meticulous specimen preparation and minimizing errors induced by plasticity or machining [92–100]. In contrast, strain tomography [87, 101–105] is a non-destructive imaging technique that reconstructs the planar distribution of the measured component of residual elastic strains within a material. Using a Radon transform-based algorithm [106, 107], strain tomography leverages diffraction data collected from multiple projections around the sample to compute the strain field. Factors such as beam collimation, diffraction geometry, and the number of projection angles influence its resolution and accuracy. Unlike the contour method, strain tomography is non-destructive, enabling repeated measurements, in-service evaluations, and validation against other techniques. However, its reliance on synchrotron or neutron diffraction facilities presents logistical and accessibility challenges. By integrating these complementary techniques, a more comprehensive understanding of residual stress distributions in complex materials can be achieved. The contour method's ability to directly provide stress fields complements the strain data from strain tomography, enabling cross-validation and refinement of strain-to-stress conversion methodologies. This synergy is particularly valuable for additively manufactured superalloys like CM247LC, where thermal gradients and microstructural anisotropy give rise to complex residual stress fields.

This study addresses a critical gap in the literature by presenting a direct comparative analysis of residual stress and strain reconstructions using both the contour method and strain tomography in LPBF fabricated CM247LC, a nickel-based superalloy known for its anisotropic microstructure and sensitivity to residual stress. While the contour method [85, 108, 109] and strain tomography [110] have each been employed independently to reconstruct planar residual stress distributions in additively manufactured parts, a systematic evaluation of their outputs on identical specimens has not previously been reported. The primary objective of this work is to assess the consistency between the stress and strain fields obtained by these two fundamentally different techniques, one destructive and direct, the other non-destructive and strain based. In particular, the study examines how well the residual stress distributions reconstructed via the contour method correspond to the elastic strain fields derived from synchrotron X-ray diffraction strain tomography, and how variations in stress gradients across different planes within the part reflect the influence of thermal history in CM247LC. The novelty of this work lies not only in its dual-method comparison, but also in its demonstration of the complementary strengths of these approaches, which together enable a more robust and spatially resolved characterization of internal stresses. This integrative methodology advances the current state of knowledge in residual stress analysis for LPBF parts by enabling cross-validation, capturing localized strain variations, and providing a validated framework applicable to other high-performance materials with complex microstructures.

## Methodology

**Fig. 1:**
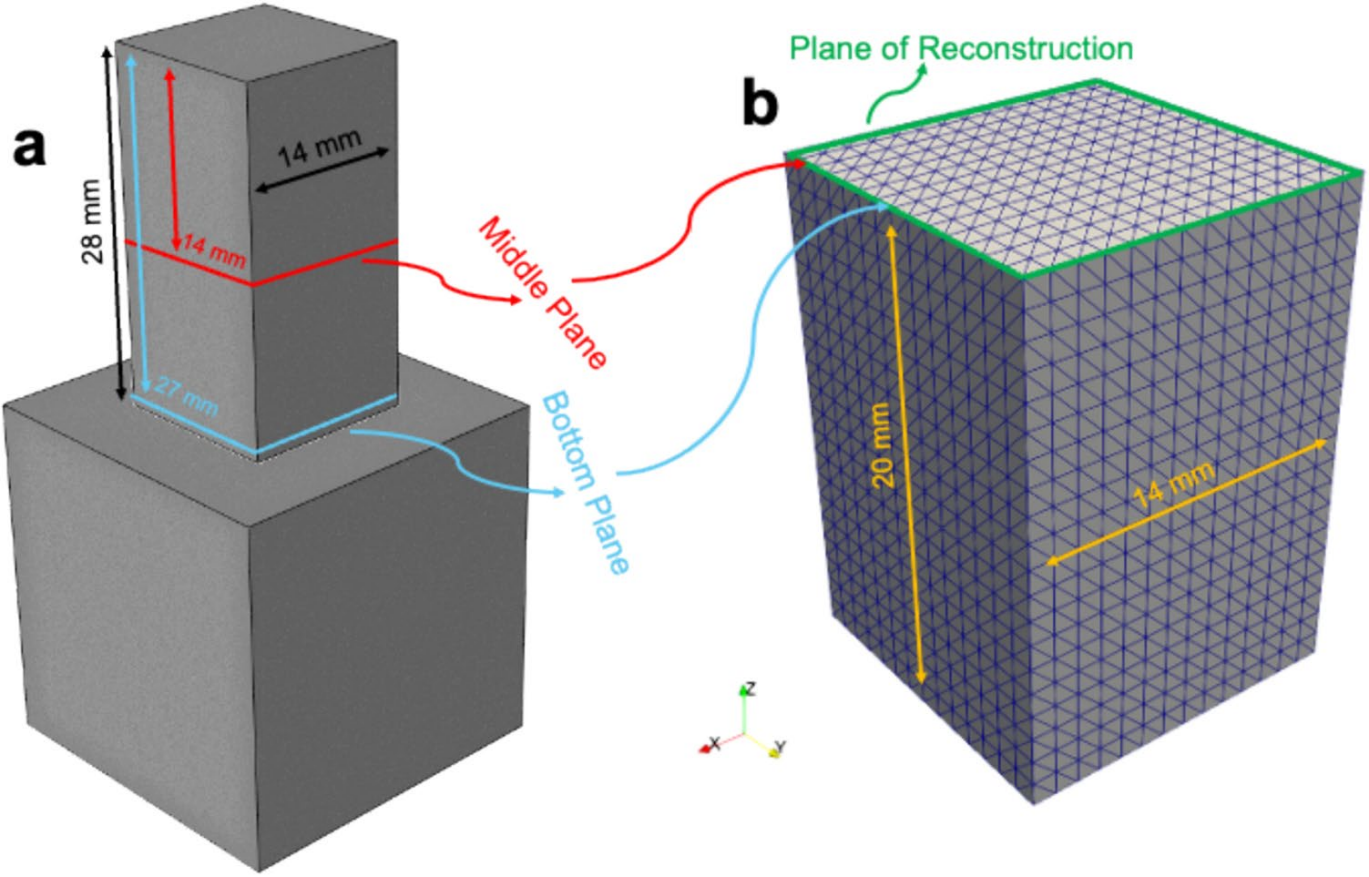
**a** Schematic representation of the CM247LC additive manufacturing specimen positioned on a steel substrate, with annotated dimensions. **b** Numerical model domain dimensions, showing the tetrahedral mesh elements and the plane of reconstruction used for contour method calculations

Three identical specimens were fabricated using LPBF technology under consistent processing parameters to ensure reproducibility and uniformity in the resulting microstructures and residual stress distributions. This study was designed to perform a blind residual stress analysis in order to eliminate potential bias arising from prior knowledge of additive manufacturing process parameters. To achieve this, specimen fabrication and residual stress reconstruction were intentionally carried out in separate laboratories. The CM247LC specimens were manufactured using LPBF at the University of Sheffield, while the residual stress analyses, via the contour method and strain tomography, were independently conducted at the University of Oxford. This separation ensured that the reconstruction of residual stress and strain fields was not influenced by expectations linked to specific process conditions. The specimens were designed with a rectangular geometry, each growing over a 14×14 mm^2^ square base plane located on a steel substrate and extending to a height of 28 mm as illustrated in Fig. 1a. These dimensions were selected to emulate the structural and thermal conditions typical of high-performance parts while accommodating the resolution requirements of the employed measurement techniques. The LPBF process inherently introduced complex residual stress fields, resulting from the localized thermal gradients and rapid solidification that are characteristic of additive manufacturing. To investigate the internal residual stresses, measurements were conducted on two specific planes within the specimens. The first plane was located 14 mm below the top surface, corresponding to the mid-height of the specimens, while the second plane was positioned 27 mm below the top surface, near the base. These planes were chosen to capture the stress variations arising from the gradient cooling rates and thermal histories across the build height.

**Fig. 2:**
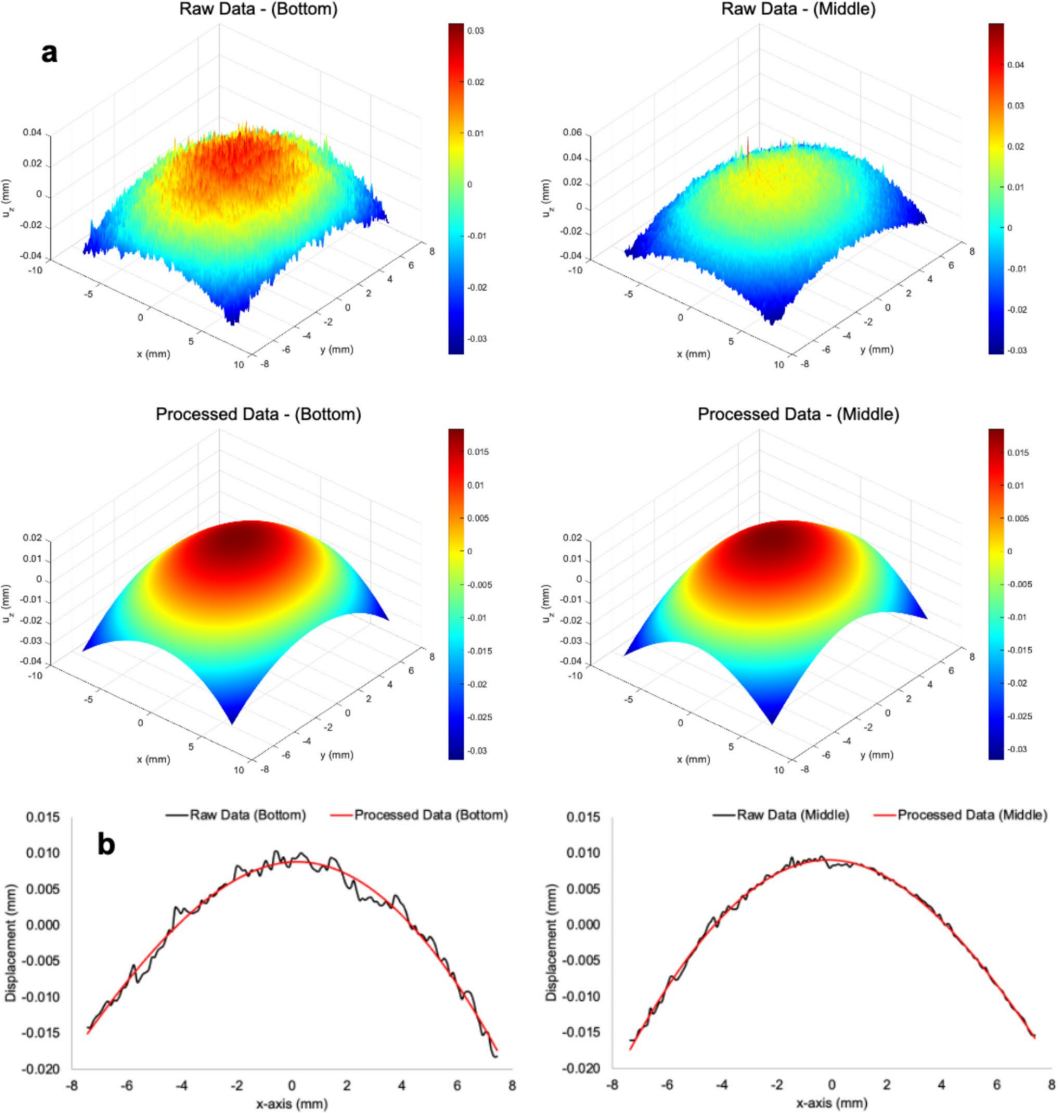
Raw and processed out-of-plane displacement data acquired from the EDM-cut surfaces at the bottom and middle planes of the CM247LC specimen, as used in the contour method reconstruction. **a** Displacement maps show peak deformation magnitudes, with a consistent distribution trend across both planes. **b** Line plots of displacements averaged along the y-axis illustrate a symmetric deformation profile, confirming the effectiveness of the smoothing algorithm in preserving true surface distortion while reducing noise artifacts

Two specimens of additively manufactured CM247LC superalloy were prepared for the contour method residual stress analysis by creating electrical discharge machining (EDM) cut surfaces. Contact profilometry was employed to collect deformation data from the cut plane, which were then processed according to the established guidelines [111] by Hosseinzadeh et al. Unlike the given procedures, profilometry data from two opposing surfaces were interpolated to a common grid, with one side rotated 90 degrees before averaging. This approach aimed to minimize the influence of experimental errors introduced during cutting, based on the assumption that the stress distribution over the square cross-section is identical along both in-plane axes. Both the raw and processed profilometry data are illustrated in Fig. 2a that is consistent with the distribution of profilometry data reported by Ahmad et al. from LPBF additive manufacturing parts with rectangular geometry [85]. A crucial step in the contour method is processing noisy profilometry data, that include smoothing, a process that heavily relies on the investigator's judgment [109]. In this study, smoothing was performed to preserve the overall deformation trends while effectively eliminating noise, as demonstrated by the line plots in Fig. 2b.

Contour method residual stress reconstructions were performed using the OxCM contour method solver [75], which applies a robust algorithm based on the static boundary condition rule. The processed profilometry data were applied to the plane of reconstruction over the domain of the numerical model, illustrated in Fig. 1b. The numerical model for the contour method reconstruction was defined over a domain measuring 14 × 14 × 20 mm^3^, resulting in a total volume of 3920 mm^3^. This domain was discretized into 3920 regularly spaced cubic cells, each with dimensions of 1 × 1 × 1 mm^3^. Within each cubic cell, six tetrahedral elements were generated using a structured meshing scheme, leading to a total of 23,520 tetrahedral elements across the model. This meshing strategy ensured consistent element distribution throughout the domain while providing sufficient resolution for accurate reconstruction of residual stress fields derived from the surface deformation measurements.

The linear elastic properties of the CM247LC superalloy were defined with a Young's modulus of 200 GPa and a Poisson's ratio of 0.3. The plane of reconstruction illustrated in Fig. 1b was strategically positioned within a computational domain extending 20 mm from the cut surface. Saint-Venant's principle [112, 113], which suggests that the influence of external loads diminishes significantly with increasing distance from the load application point, was applied to determine this domain depth as described in the scientific paper of the OxCM contour method solver [75]. Consequently, the depth of the computational domain was selected to ensure negligible residual stress levels beyond the reconstructed region. To ensure negligible residual stress levels beyond the reconstructed region, a rigorous extrusion depth analysis was performed. This analysis involved incrementally increasing the domain depth and evaluating the stress decay behaviour. The computational domain was chosen to have the depth for which reconstruction stresses reached a negligible level, and also accounts for the constraints and boundary effects of the experimental setup. Furthermore, the static boundary condition rule was applied by implementing processed displacement data to the plane of reconstruction and symmetry boundary condition on the back end of the domain, parallel to the plane of reconstruction where the residual stresses completely vanish.

In this study, synchrotron X-ray diffraction strain tomography was employed to non-destructively and precisely map residual elastic strains. The approach follows the methodology outlined by Korsunsky et al. [87], utilizing the filtered-back projection algorithm (iradon) implemented within the MATLAB® programming environment. All the available filtering techniques provided by the iradon function were tested, but no significant differences in the magnitude or distribution of the mapped strains were observed, leading to the use of the default Ram-Lak filter setting.

The synchrotron X-ray diffraction experiments were conducted at the ID15A beamline of the European Synchrotron Radiation Facility (ESRF) in Grenoble, France. The CM247LC superalloy additive manufacturing part was mounted on a sample stage capable of triaxial translation and rotation around the vertical z-axis. A monochromatic incident beam, collimated to 100×100 µm^2^ beam size with a photon energy of 100 keV, passed through the specimen along the gauge volume with an exposure time of 4 seconds and scattered to form diffraction cones collected by a two-dimensional Perkin-Elmer large-area detector. Calibration was performed using stress-free CeO₂ to determine the exact radiation energy and sample-detector distance.

Scans were conducted over the same sampling planes as those used in the contour method, illustrated in Fig. 1a, covering a 180-degree angular range with a step size of 5 degrees starting from 0-degree. A total of 115 beam spots spanned a translation range of 23.0 mm, exceeding the specimen's diagonal size of 19.8 mm. Diffraction rings resulting from Bragg scattering at an angle of 2θ were observed on the detector. The diffraction data composed of these rings was analysed to calculate their apparent radius and corresponding residual elastic strains through trigonometric averaging, utilizing the extended caking method as described in the scientific paper of this method [114] using the exCaking console application.

## Results

**Fig. 3:**
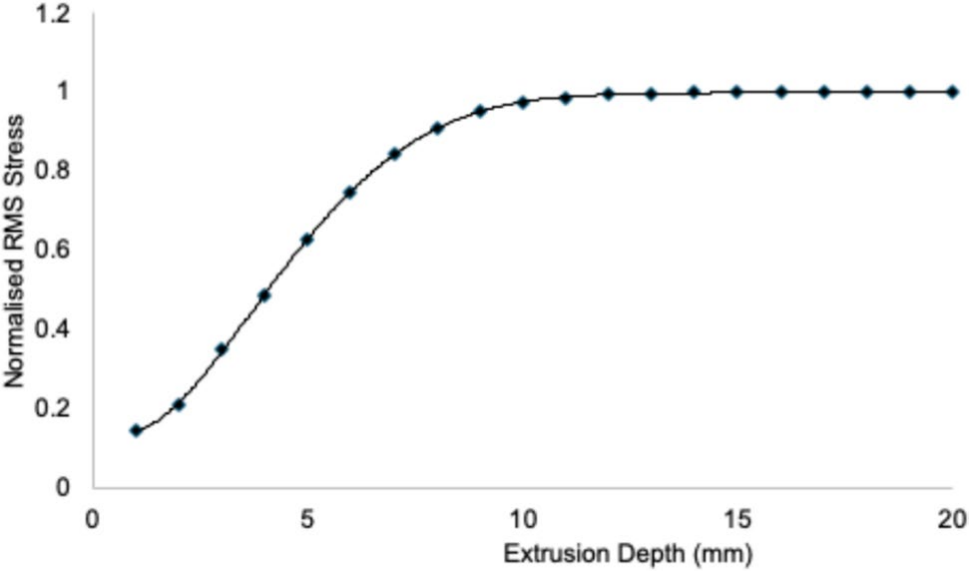
RMS residual stress as a function of extrusion depth used in the contour method finite element reconstruction. Stress levels stabilize beyond a depth of 15 mm. This saturation confirms that a 20 mm extrusion depth is sufficient to capture the complete stress field while minimizing boundary effects, in accordance with Saint-Venant's principle

The variation in root mean squared stress (RMS) with respect to extrusion depth, as shown in Fig. 3, highlights the stabilization of residual stress values beyond an extrusion depth of 15 mm. This negligible change in RMS stress at greater depths indicates that stress saturation is achieved within this region, validating the adequacy of the numerical domain for accurately capturing the residual stress state. Based on these observations, the residual stress analysis was conducted using a numerical domain extruded to a depth of 20 mm, as illustrated in Fig. 1a. The chosen domain depth ensured that the stress field was fully resolved while minimizing computational overhead. This approach also accounted for Saint-Venant's principle [75], confirming that the influence of boundary conditions at the domain's extremities did not affect the reconstructed stress fields. The results further demonstrate the robustness of the methodology, with stress gradients near the surface and within the region of interest accurately captured. This analysis provides critical insights into the internal stress distributions of the additively manufactured CM247LC superalloy, enabling a comprehensive understanding of the effects of the LPBF process on residual stress evolution.

**Fig. 4:**
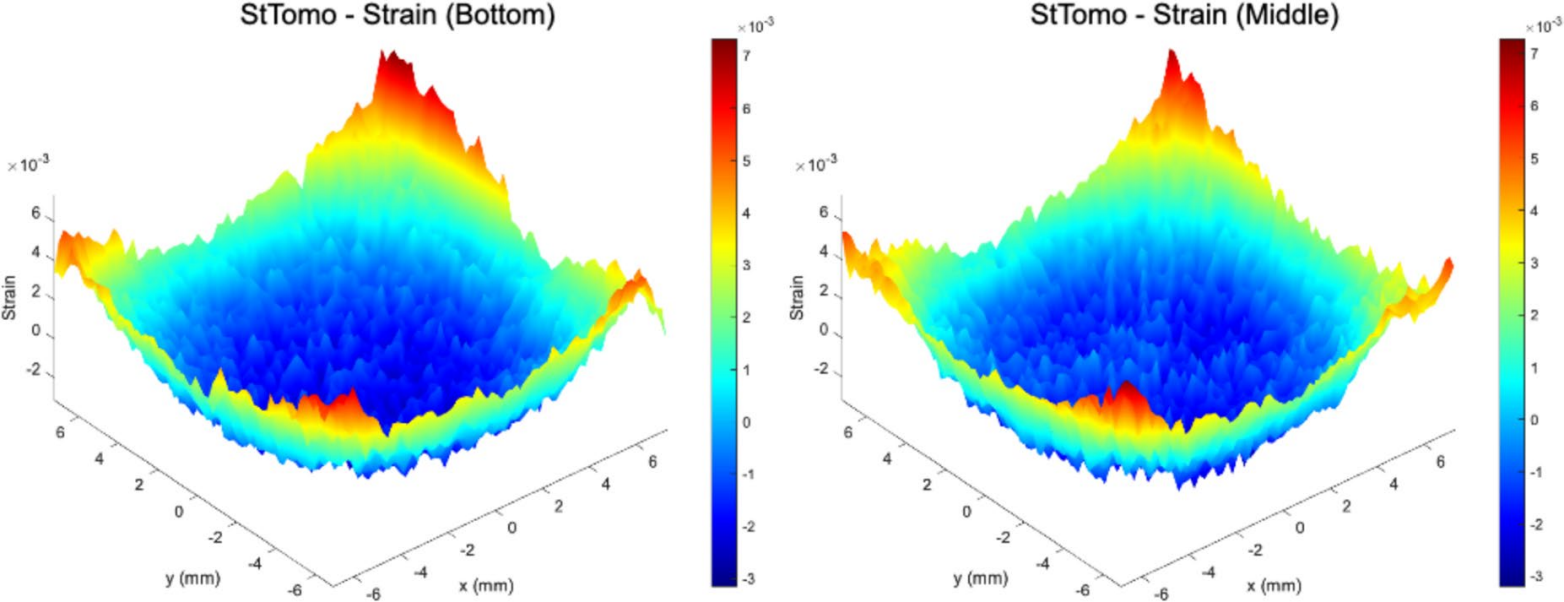
Raw residual elastic strain distributions reconstructed from synchrotron X-ray diffraction data using strain tomography at the bottom and middle planes. Compressive strains reaching up to -0.003 were observed in central regions, surrounded by tensile strains of up to 0.007 near the edges. A pronounced increase in central compressive strain was detected at the bottom plane compared to the middle plane, highlighting the evolution of internal stress states along the build height

Strain tomography results, as depicted in Fig. 4, reveal a distinct distribution of residual strains across the investigated planes. The central regions exhibit compressive strains, which are surrounded by tensile strains along the edges. While the overall range of strain magnitudes is consistent across the planes, notable differences are observed in the central regions. The middle plane demonstrates a reduced magnitude of compressive strains compared to the other plane, whereas the bottom plane exhibits a progressive increase in compressive strain magnitude toward the centre. This trend suggests a complex evolution of strain states influenced by the additive manufacturing process and subsequent thermal gradients. The strain tomography calculations were derived using unprocessed results from the extended caking strain analysis, ensuring the representation of realistic, raw strain values. Conversely, the contour method inherently requires processing of experimental deformation data, leading to strain and stress distributions with smooth profiles. To enable a meaningful comparison, strain tomography results were subjected to post-processing, by applying the same smoothing procedures and parameters used for profilometry data, to achieve a comparable level of smoothness in strain distribution. This step ensures consistency in the analysis framework, facilitating a robust comparison between the strain tomography and contour method reconstructions.

**Fig. 5:**
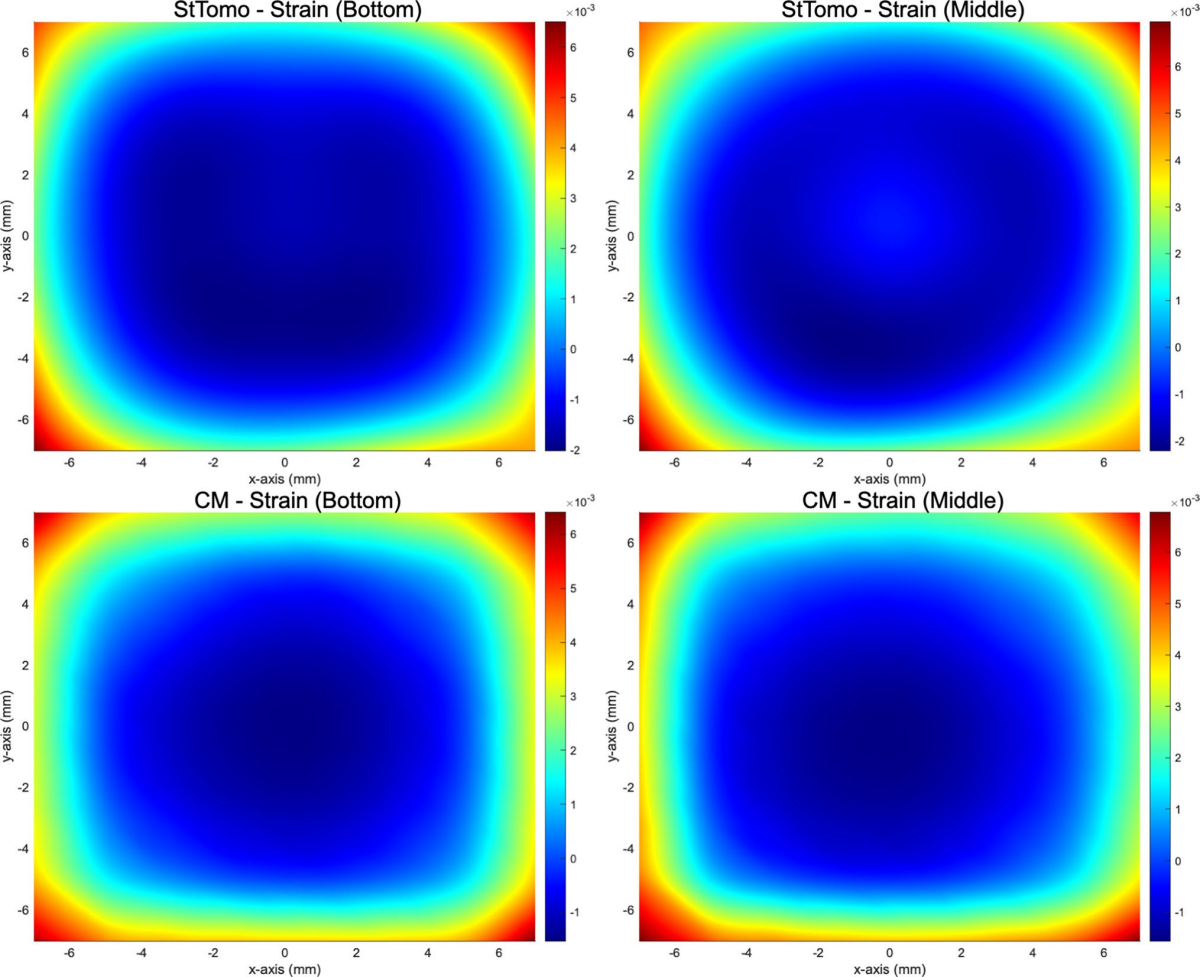
Comparison of smoothed residual elastic strain fields reconstructed via the contour method and strain tomography for the bottom and middle planes. Both methods reveal tensile strain peaks exceeding +0.006 at the specimen edges and central compressive strains up to -0.002. While the contour method exhibits smoothed strain transitions, strain tomography captures localized strain heterogeneities, demonstrating its superior sensitivity to subtle spatial variations within the additively manufactured part

A comparison of the smoothed residual elastic strain distributions obtained from strain tomography and the contour method, as presented in Fig. 5, reveals a consistent trend in the overall distribution of strains. Both methods demonstrate the presence of tensile residual elastic strains concentrated at the edges of the specimen, counterbalanced by compressive strains towards the central regions. Notably, the strain tomography effectively captures localized variations in the central region, providing detailed insights into the strain distribution within this critical area. In contrast, the contour method depicts a progressive increase in compressive strain magnitude towards the centre, suggesting a more homogenized representation of the strain field. These differences can be attributed to several factors inherent to each technique. First, the contour method, which relies on surface deformation measured after EDM-cutting, is sensitive to cutting-induced artifacts and noise in profilometry data. Although smoothing procedures mitigate these effects, they may also suppress fine-scale variations. In contrast, strain tomography, based on diffraction measurements and Radon transform algorithms, offers higher sensitivity to localized strain variations, especially in complex microstructural regions, but is limited by beam size and projection resolution. Additionally, the contour method assumes isotropic, linear elastic behaviour and applies a symmetry-based smoothing strategy, whereas strain tomography directly reconstructs strain fields from diffraction data, with minimal post-processing. Accordingly, methodological differences result in variations in spatial resolution and reconstruction fidelity. These findings draw attention to the sensitivity of the contour method to procedural nuances while highlighting the capability of strain tomography to capture subtle variations in strain fields.

An important advantage demonstrated by both the contour method and strain tomography in this study is their ability to evaluate residual stresses and residual elastic strains without relying on assumptions regarding material plasticity. The contour method derives residual stresses from elastic surface deformations measured after cutting, and the subsequent finite element reconstruction is performed under the assumption of linear elasticity. Since the deformation used in the reconstruction occurs upon stress release and is elastic in nature, the method inherently avoids any need to model prior plastic behaviour. Similarly, strain tomography reconstructs internal elastic strain fields from diffraction-based measurements, which directly probe the lattice spacing in a stress-free reference state. These measurements reflect purely elastic responses of the material and are converted to stress using known elastic constants, again avoiding any dependence on plastic deformation models. Consequently, both methods offer reliable stress analysis in materials like CM247LC, where plastic behaviour is difficult to characterize due to its high strength and complex microstructure.

**Fig. 6:**
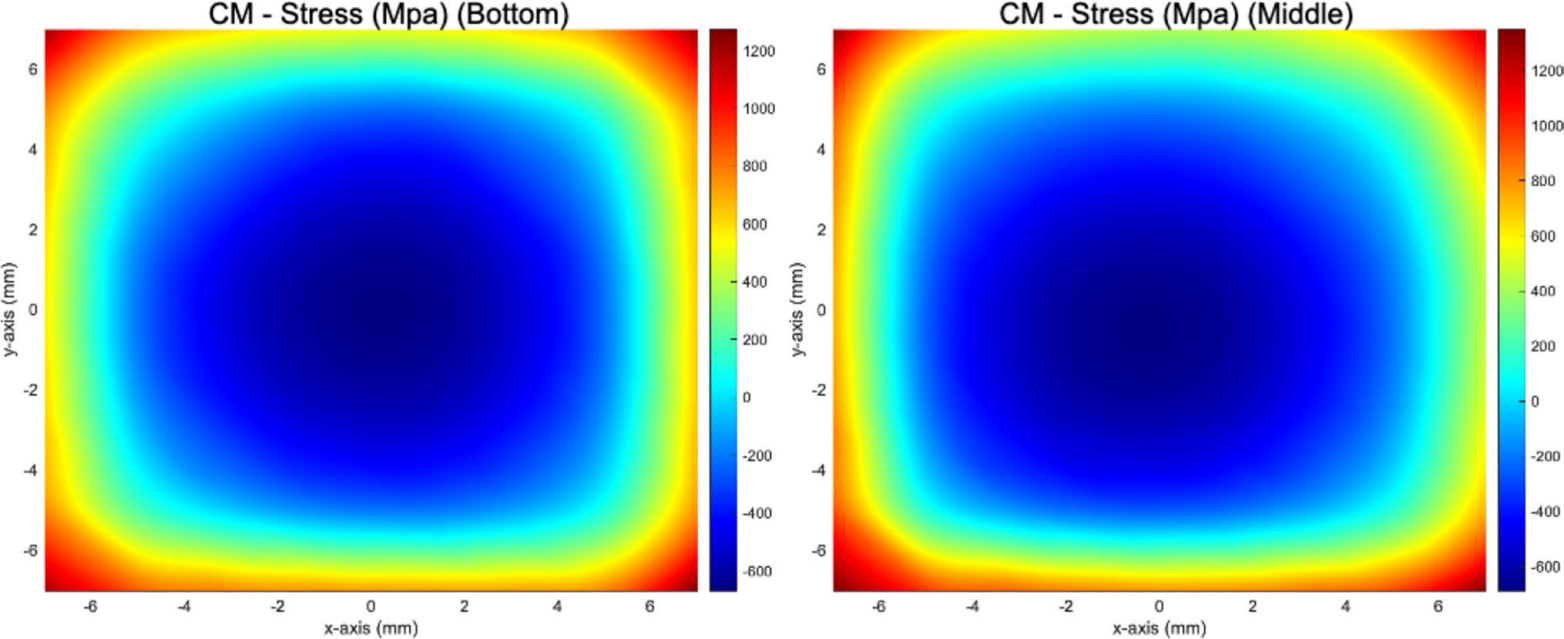
Reconstructed residual stress distributions in the zz-direction obtained using the contour method for the bottom and middle planes of the CM247LC specimen. Maximum tensile stresses of up to +1300 MPa were identified along the specimen edges, while central regions showed compressive stresses as low as -600 MPa. The middle plane exhibited higher peak tensile stresses than the bottom plane, indicating an upward shift in stress magnitudes due to thermal gradients during the LPBF process

A comparative analysis of residual stress distributions between the middle plane and the bottom plane revealed similar qualitative patterns, as illustrated in Fig. 6. However, the tensile residual stresses were observed to reach higher magnitudes in the middle plane, indicating variations in stress concentration likely induced by thermal gradients and cooling rates during the LPBF process. Notably, the results show that the residual stresses exhibit an identical spatial distribution to the residual elastic strains obtained via strain tomography. This observation highlights the intrinsic coupling between residual stress and strain fields in these parts, governed by the material's elastic properties.

The residual stress magnitudes observed in this study, particularly the peak tensile stresses approaching 1300 MPa as shown in Fig. 6, are consistent with values reported in the literature for CM247LC. Alhuzaim et al. [115] demonstrated that CM247LC thin-wall structures fabricated via direct laser deposition can achieve ultimate tensile strengths up to 1120 MPa, depending on processing parameters and geometry. Furthermore, Uzun et al. [110] showed that the zz-component of residual stress in LPBF-manufactured CM247LC can reach comparable magnitudes, confirming the plausibility of high tensile stress accumulation in such parts. These findings support the reliability of the residual stress quantification obtained through the contour method in the current study. It is acknowledged that the observed stress magnitudes may approach or slightly exceed the nominal yield strength typically expected for CM247LC. However, it is important to note that the stress reconstruction methods employed, both the contour method and strain tomography, are based on the assumption of linear elastic behaviour. The conversion from strain to stress is conducted using elastic constants, without invoking yield criteria or plastic deformation models. While the von Mises criterion could, in principle, be used to assess yielding by evaluating the full stress tensor, such an analysis requires knowledge of all six independent stress components. In this study, only selected components, primarily the normal stress in the zz-direction, were reconstructed, precluding a complete tensor-based yield assessment. Nevertheless, the good agreement with literature-reported values suggests that the stress results presented here are physically meaningful within the elastic framework of the employed methods.

The findings of this study align with existing literature on residual stress patterns in additively manufactured CM247LC superalloy parts, while also offering new insights. Consistent with previous research, results indicate that tensile residual stresses predominantly occur near the part's surface, with compressive stresses in the core [85, 110]. This distribution is attributed to thermal gradients and the layer-by-layer nature of the additive manufacturing process. For instance, a study on laser-directed energy deposition of CM247LC reported tensile stresses reaching beyond 1300 MPa near the surface, decreasing with depth, which is in line with observations [27]. However, present study also reveals that the magnitude of these residual stresses can be influenced by process parameters, such as inter-layer dwell times and scanning strategies. Notably, the application of inter-layer dwell times has been shown to significantly reduce residual stresses [29], a finding that warrants further investigation to optimize additive manufacturing processes for CM247LC.

The observed congruence between stress and strain distributions not only underscores the fidelity of both reconstruction techniques but also provides insights into the mechanisms governing residual stress development in additively manufactured CM247LC. The agreement between the methods confirms that elastic deformation captured via surface profiling and lattice strain measured via diffraction represent two facets of the same residual stress phenomenon. Notably, the consistency in stress and strain spatial gradients supports the assumption that plastic deformation during the EDM-cutting process was minimal or localized, reinforcing the validity of a linear elastic approximation for post-process analysis. Furthermore, while the contour method emphasizes global stress field patterns due to its spatial smoothing, strain tomography revealed subtle local strain anomalies, especially near the transition between compressive core and tensile periphery, that may reflect microstructural heterogeneity or slight deviations in thermal history across the build height. These features would be challenging to resolve using a single technique alone. Importantly, the stress–strain field congruence across two distinct planes suggests that the residual stress architecture is largely governed by systematic thermal gradients intrinsic to LPBF, rather than stochastic defects or isolated anomalies. This spatial regularity points to the possibility of predictive modelling based on thermal simulation and microstructure-informed elasticity, which could guide future process optimization. Together, these results demonstrate the complementary power of destructive and non-destructive techniques in validating the internal mechanical state of complex superalloy builds.

The results of this study have significant implications for the design and post-processing strategies of CM247LC parts fabricated via LPBF. The detailed comparison between the contour method and strain tomography, as illustrated in the revised Fig. 2 through Fig. 6, reveals consistent patterns of tensile stresses accumulating near the part edges and compressive stresses concentrated in the core. These insights suggest that residual stresses in CM247LC are strongly influenced by thermal gradients and geometric constraints inherent to the LPBF process. Consequently, optimizing build orientation to minimize steep thermal gradients, such as orienting critical surfaces away from high-tension zones, could be an effective strategy for stress mitigation. Furthermore, the spatial mapping of residual strain evolution across the build height indicates that heat treatment protocols could be tailored to address localized stress concentrations, particularly in mid-height regions where peak tensile stresses were observed. The ability of strain tomography to capture subtle strain heterogeneities also offers a pathway for validating and refining heat treatment cycles aimed at homogenizing internal stress fields. Overall, the combined use of destructive and non-destructive reconstructions in this study provides a validated framework that can  guide predictive thermo-mechanical models and process optimization for improved structural reliability of CM247LC parts in high-performance applications.

In interpreting the results of this study, it is essential to acknowledge the inherent limitations associated with both the contour method and strain tomography, as these may influence the accuracy of the residual stress measurements. The contour method operates under the assumption that material removal during cutting induces purely elastic deformation; however, when residual stresses approach or exceed the material's yield strength, plastic deformation can occur, leading to potential inaccuracies in stress reconstruction. Additionally, deviations in the cutting path, even if minor, can affect the precision of the measured surface deformations, thereby impacting the stress calculations. To mitigate these issues, future work could involve implementing incremental cutting techniques to minimize plasticity effects and employing high-precision cutting equipment to ensure consistent cut paths. Regarding strain tomography, limitations include assumptions of uniform material properties and potential challenges in capturing fine-scale strain variations due to resolution constraints. Addressing these limitations may involve integrating higher-resolution detectors and developing advanced reconstruction algorithms that account for material heterogeneities. Recognizing and addressing these limitations are crucial for enhancing the reliability of residual stress measurements in additively manufactured CM247LC parts.

## Conclusions

This study presented a rigorous comparative analysis of residual stress and strain distributions in LPBF fabricated CM247LC superalloy parts, using two advanced yet fundamentally distinct experimental techniques: the contour method and synchrotron X-ray strain tomography. The integration of these complementary approaches enabled a validated, high-resolution reconstruction of internal mechanical fields in a high-performance nickel-based superalloy with complex thermal history and microstructural features.

The contour method provided stress reconstructions based on elastic surface displacements induced by EDM-cutting, while strain tomography offered non-destructive strain field reconstructions through diffraction-based tomographic imaging. Both methods independently revealed a characteristic residual stress architecture, featuring high tensile stresses localized near specimen edges and compressive cores, with stress magnitudes exceeding +1300 MPa and descending to -600 MPa. Importantly, strain and stress distributions exhibited strong spatial congruence, confirming the reliability of linear elastic assumptions and indicating that plasticity during processing or measurement was minimal.

Differences between the two methods, most notably the localized strain variations captured more distinctly via strain tomography, highlight the methodological sensitivities of each technique. The fidelity of strain tomography in capturing local heterogeneities complements the global robustness of the contour method, offering a pathway to improve the resolution and reliability of residual stress characterizations through multimodal integration.

Crucially, the spatial trends observed across build height emphasize the role of thermal gradients and process parameters in shaping residual stress fields. These findings provide actionable insights for optimizing LPBF process strategies, such as build orientation, scan path design, and inter-layer dwell times. Moreover, the results suggest that post-processing treatments, including spatially targeted heat treatments, can be more effectively designed when informed by high-fidelity stress maps.

While the present study advances residual stress characterization capabilities, it also acknowledges certain limitations, including assumptions of linear elasticity and the resolution constraints inherent in each method. Future research should aim to incorporate anisotropic elasticity models and leverage higher resolution tomography.

Overall, this work establishes a robust framework for the validation and interpretation of internal stress states in additively manufactured superalloys. The demonstrated synergy between destructive and non-destructive methods offers a valuable toolset for the design, qualification, and lifecycle management of critical parts in aerospace and energy applications.

## Data Availability

The synchrotron X-ray diffraction data supporting the findings of this study will become openly available in the data portal of ESRF in 2025 at doi.org/10.15151/ESRF-ES-751303497 at the end of an embargo period.
